# Medical Students' Stress Levels and Sense of Well Being after Six Weeks of Yoga and Meditation

**DOI:** 10.1155/2016/9251849

**Published:** 2016-12-07

**Authors:** Lona Prasad, Aneesha Varrey, Giovanni Sisti

**Affiliations:** Department of Obstetrics and Gynecology, New York Presbyterian Hospital, Weill Cornell Medical Center, 525 East 68th Street, Suite J-130, New York, NY 10065, USA

## Abstract

*Objective*. To determine the effect of six weeks of yoga and meditation on medical students' levels of perceived stress and sense of wellbeing prior to taking their exams.* Methods*. We conducted a prospective case-control study of first-through-third-year medical students at our academic institution, measuring levels of perceived stress and sense of wellbeing before and after a six-week yoga and meditation intervention. Questionnaires used for evaluation included the perceived stress scale (PSS) and self-assessment surveys (SAS). The postintervention surveys were completed on the day of the students' written exams.* Results*. A total of thirteen women and fourteen men participated. Median age was 28 (24 yrs–32 yrs). 48.1% were Caucasian, 7.4% Black, 11.1% Hispanic, 11.1% Asian, and 22.2% other. Paired *t*-tests showed a statistically significant reduction in perceived stress (18.44 versus 14.52; *p* = 0.004) after the six-week yoga and meditation program. After the yoga intervention, self-assessment survey results showed a significant improvement in feelings of peace, focus, and endurance. Improvements in happiness, positivity, personal satisfaction, and self-confidence were also seen. An improvement in unsubstantiated parameters such as patience and fatigue was observed.* Conclusion*. Yoga and meditation may be effective in reducing stress levels and improving aspects of personal wellbeing in medical students.

## 1. Introduction

Stress amongst medical students is experienced in response to a range of occupational stimuli. These include sacrificing time spent with loved ones, acquiring sizable financial debt, and experiencing sexual harassment or professional abuse. Dealing with issues of human suffering and mortality can be emotionally challenging. Finally, working to master increasing amounts of information in limited time periods can contribute to academic stress [1]. Investigators have reported that the need to perform well on exams and preparing for and taking exams were the most stressful situations that students experienced in medical school [2]. Beyond certain levels, however, continuous exposure to stress may negatively impact the physical and mental health of students.

It has been shown that medical students have a high rate of deterioration in quality of life due to work hours and hazardous work related behavior patterns. In the United States, approximately 50% of medical students experience burnout, 25% have depression, and many suffer from chronic anxiety [3, 4]. Perpetual distress adversely affects the development of students' knowledge, skills, and professionalism. Students' ability to establish good relationships with patients has been compromised resulting in feelings of inadequacy. This has been associated with dissatisfaction which continues into residency and future clinical practice [5].

Mind body interventions are increasingly being used in the general population to assist with stress reduction. Mental silence-oriented meditation has been shown to be a safe and effective strategy for dealing with work stress and depressive feelings in full time workers [6]. Patients undergoing IVF report high levels of depressive symptoms, anxiety, and distress. Yoga has been found to improve the overall quality of life related to infertility and to reduce general anxiety and depression over time [7]. Yoga and meditation techniques have also been found to reduce performance anxiety and mood disturbance in young professional musicians [8]. There is evidence which suggests that meditation-based stress management practices reduce stress and enhance forgiveness among college undergraduates [9].

Our study focuses on yoga, an ancient Indian system of philosophy and practice. Modern yoga practice has been influenced by the “Eight-Limbed Path” of yoga, as described by Patanjali in The Yoga Sutras in 200 CE [10]. This text along with the Hatha Yoga Pradipika written in the 15th century CE suggests that one may gain physical, emotional, and spiritual health through the practices of yoga [11]. Hatha yoga practice incorporates breath awareness with bodily postures requiring focus and improving strength, flexibility, and balance. Other aspects of yoga include pranayama or “breath control” exercises which intentionally alter one's breathing pattern to help achieve mindful concentration. Meditation is a state which enables one to focus on the present moment, leading to a state of thoughtless awareness [12].

There are limited reports of structured yoga programs used in medical schools to promote students' wellbeing and reduce stress levels. A mindfulness based stress reduction (MBSR) intervention has been shown to improve perceived stress and self-compassion and promote self-awareness, self-reflection, and self-care in first-year medical students [13, 14]. Similar to our focus on yoga, a pilot study done in Montreal showed that a sixteen-week yoga intervention may be effective in decreasing stress and improving general wellbeing in first-year medical students [15]. Also, a randomized controlled trial conducted in India showed that a yoga intervention reduced levels of anxiety in first-year medical students prior to taking exams [16]. The primary aim of our study was to determine whether incorporating the practice of yoga into the first-through-third-year medical school curriculum for a six-week period would enable students to reduce perceived stress and experience an improvement in personal wellbeing prior to taking their exams.

## 2. Materials and Methods

This was a prospective case-control study undertaken between October 2013 and June 2015 at New York Presbyterian Hospital, Weill Cornell Medical School, in New York. The study was approved by the Weill Cornell Medicine Research and Ethics Committee. First-through-third-year medical students regardless of age, gender, or ethnicity were recruited by email prior to beginning either their six-week biological sciences course (first and second years) or clinical rotation (third years). Upon response, they were asked to complete an intake form and physical activity readiness questionnaire (PAR-Q) as follows:

*Physical Activity Questionnaire (PAR-Q)*

Identifier Code: —
Age: —
Date: —

Please read the following questions carefully and check (X) the appropriate answer. Answer all questions honestly and to the best of your ability.

1. Has your doctor ever said that you have a heart condition (had a stroke, heart attack, or heart surgery) and/or that you should only do physical activity recommended by a doctor?
  Yes: —
  No: —
2. Do you feel pain in your chest when you do physical activity?
  Yes: —
  No: —
3. In the past month, have you had chest pain when you were not doing physical activity?
  Yes: —
  No: —
4. Do you lose your balance because of dizziness or do you ever lose consciousness?
  Yes: —
  No: —
5. Have you ever been told by a doctor that you have bone, joint or muscle problems that could be made worse by physical activity?
  Yes: —
  No: —
6. Do you have a diagnosed illness that could be made worse by physical activity?
  Yes: —
  No: —
7. Is your doctor currently prescribing medication for your blood pressure or heart condition?
  Yes: —
  No: —
8. Are you pregnant?
  Yes: —
  No: —
9. Do you know of any other reason why you should not do physical activity?
  Yes: —
  No: —

*Fitness Participation Agreement*. I have answered the questions above to the best of my ability and affirm that my physical condition is good and I have no known conditions that would prevent me from participation. I acknowledge that participation is at my own pace and comfort level and that I may discontinue my participation at any time. Furthermore, I agree to self-determine my exertion through good judgement and to discontinue any activity that exceeds my personal limitations.

Signature of Participant: —
Date: —

Participants were excluded if they had engaged in greater than one year of weekly yoga and if they had practiced weekly in the three months preceding the start date of the study. They had to meet wellness criteria determined by the physical activity readiness questionnaire and be able to attend and participate in the classes. Consent was obtained on initial contact.

All participants were required to attend one hour biweekly Hatha yoga classes for six weeks consisting of forty minutes of asanas (postures), ten minutes of pranayama (breathing exercises), and ten minutes of meditation. Students participated for twelve hours in total. Classes took place in the medical students' lounge, in the evenings on campus. Each session was conducted by a 500-hour certified yoga instructor with over fifteen years of experience. Classes were taught at an open level providing options for modifications based on the individual's requirements.

Data was obtained from participant self-reported questionnaires, specifically the perceived stress scale (PSS) and self-assessment surveys (SAS), respectively, as follows:

*Perceived Stress Scale Survey*. The questions in this scale ask you about your feelings and thoughts during the last month. In each case, you will be asked to indicate by circling how often you feel or thought a certain way.

Identifier Code: —
Date: —
Age: —
Gender (Circle):
  M
  F
Other: —
0 = Never
1 = Almost Never
2 = Sometimes
3 = Fairly Often
4 = Very Often
In the last month, how often have you been upset because of something that happened unexpectedly?
  0
  1
  2
  3
  4
In the last month, how often have you felt that you were unable to control the important things in your life?
  0
  1
  2
  3
  4
In the last month, how often have you felt nervous and stressed
  0
  1
  2
  3
  4
In the last month, how often have you felt confident about your ability to handle your personal problems?
  0
  1
  2
  3
  4
In the last month, how often have you felt that things were going your way?
  0
  1
  2
  3
  4
In the last month, how often have you found that you could not cope with all the things that you had to do?
  0
  1
  2
  3
  4
In the last month, how often have you been able to control irritations in your life?
  0
  1
  2
  3
  4
In the last month, how often have you felt that you were on top of things?
  0
  1
  2
  3
  4
In the last month, how often have you been angered because of things that were outside of your control?
  0
  1
  2
  3
  4
In the last month, how often have you felt difficulties were piling up so high that you could not overcome them?
  0
  1
  2
  3
  4

*Self Assessment Survey*

Identifier Code: —
Date: —

I have:

1. a sense of happiness (Strongly Disagree/Disagree/Neither Agree or Disagree/Agree/Strongly Agree)
2. a peaceful feeling (Strongly Disagree/Disagree/Neither Agree or Disagree/Agree/Strongly Agree)
3. the ability to be focused (Strongly Disagree/Disagree/Neither Agree or Disagree/Agree/Strongly Agree)
4. increased endurance (Strongly Disagree/Disagree/Neither Agree or Disagree/Agree/Strongly Agree)
5. a positive outlook (Strongly Disagree/Disagree/Neither Agree or Disagree/Agree/Strongly Agree)
6. personal satisfaction (Strongly Disagree/Disagree/Neither Agree or Disagree/Agree/Strongly Agree)
7. self confidence (Strongly Disagree/Disagree/Neither Agree or Disagree/Agree/Strongly Agree)
8. patience (Strongly Disagree/Disagree/Neither Agree or Disagree/Agree/Strongly Agree)
9. fatigue (Strongly Disagree/Disagree/Neither Agree or Disagree/Agree/Strongly Agree)

These were completed at baseline prior to commencing their academic and clinical rotations and at six weeks after the yoga intervention, just before taking their end of rotation written exams. The perceived stress scale is a ten-item questionnaire that poses general questions allowing users to respond according to their personal stressors. This results in a “global” measurement of stress. Questions are based on a five-point Likert scale. Scores range from zero to forty with higher scores indicating higher levels of perceived stress. Score points around thirteen are average. Twenty or higher is an indicator of moderate to high stress [17]. We created the self-assessment survey. This is a nine-item questionnaire using a five-point Likert scale to assess medical students' general sense of wellbeing. Questions included in the self-assessment survey measured happiness, peace, focus, endurance, positivity, personal satisfaction, self-confidence, patience, and fatigue. The first seven of these components are synonymous with those incorporated as part of the established fourteen-item Warwick-Edinburgh mental wellbeing scale validated on a student population [18]. The latter scale includes correlating phrases such as “I've been feeling cheerful,” “I've been relaxed,” “I've been thinking clearly,” “I've had energy to spare,” “I've been feeling optimistic about the future,” “I've been feeling good about myself,” and “I've been feeling confident.” Although the other two components, patience and fatigue, are not used as part of the Warwick-Edinburgh mental wellbeing scale, we chose to include these parameters as part of our self-assessment survey with the interest of increasing our scope of comparison understanding that these characteristics are relevant to performance in medical school. Similar items have been previously incorporated into a subjective assessment tool used in an Indian randomized controlled yoga trial. In this study, anxiety levels and general wellbeing were measured in first-year medical students before and after a yoga intervention prior to taking their exams. Survey parameters to gain students' feedback included rating sense of wellbeing, feeling of relaxation, ability to concentrate, feeling refreshed, level of self-confidence, task efficiency, irritability levels, stamina, exhaustion, appetite, optimistic outlook, headache/body ache, and interpersonal relationships [16]. Irritability levels and exhaustion are comparable descriptive terms to patience and fatigue. The latter were included as part of our self-assessment survey and have been displayed in our graph; however, we acknowledge that these are not substantiated parameters. In order to maintain a consistent and validated measurement, only scores for items synonymous with those used in the Warwick-Edinburgh mental wellbeing scale, that is, happiness, peace, focus, endurance, positivity, personal satisfaction, and self-confidence, are reliable in our analysis. Scores of our self-assessment survey including these seven items ranged from zero to 28 with higher numbers indicating a greater sense of wellbeing.

The Statistical Program for the Social Sciences (SPSS), version 22.0 (IBM), was used for data analysis. The normality of the data was checked with the Shapiro-Wilk test. Paired samples *t*-test comparisons were performed on the perceived stress scale survey scores before and after the yoga intervention.

## 3. Results

A total of 34 medical students volunteered to participate in the study. One was excluded because of her regular yoga practice. Six dropped out before completing the six-week yoga program. The reasons given included being too busy with medical school responsibilities or unwell. Twenty-seven medical students completed the six-week yoga program, answered all the questionnaires, and therefore were included in the analyses. A total of thirteen women and fourteen men participated. The median age was 28 years (24–32). Eight (29.6%) were in their third year. Other participants included fifteen (55.5%) second-year and four (14.8%) first-year students. Forty-eight percent of the study sample were Caucasian, 7.4% were Black, 11.1% were Hispanic, 11.1% were Asian, and 22% identified themselves as other. Twenty (74%) students had previous experience with yoga, but none had practiced regularly.

Eleven (41%) students had previous experience with meditation. Twenty-three (85%) reported involvement between one and five hours of physical exercise per week at the onset of the yoga study. Five (18.5%) participants reported taking medications.

**Figure 1:**
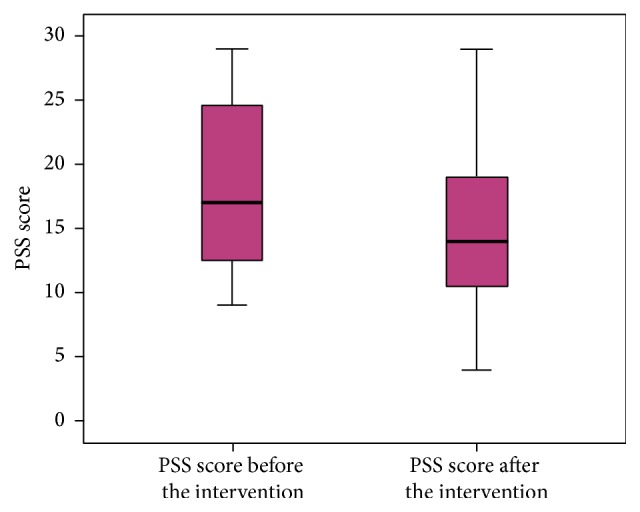
Perceived stress scale (PSS) graph.

**Figure 2:**
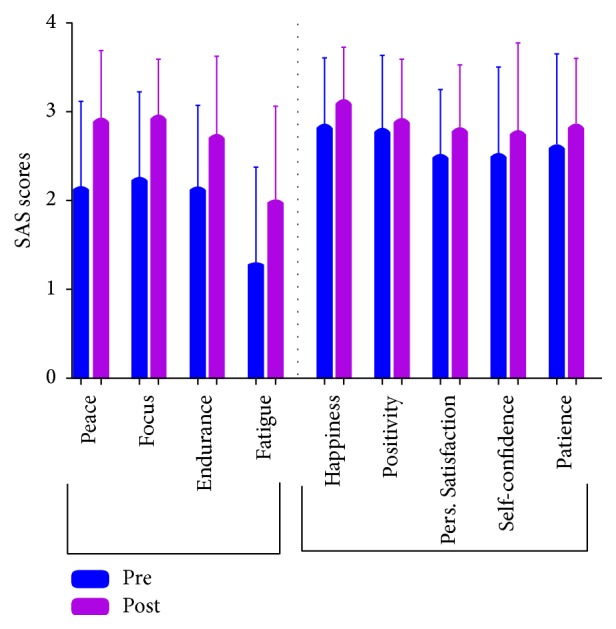
Self-assessment survey (SAS) graph.

The perceived stress scale showed a statistically significant reduction in stress levels from baseline compared to six weeks after the yoga intervention (18.44 versus 14.52; *p* = 0.004) (Figure 1). The mean baseline perceived stress score in our study population was 18.44 which is less than the scores documented in the literature for student populations (23.7 and 23.2) [17]. However, twelve of the 27 (44.4%) students were considered to be moderately to highly stressed at baseline with a score ≥20. Results of the self-assessment survey showed that there were significant improvements in feelings of peace, focus, and endurance. The increase in survey scores ranged between one and four points for each of these parameters from baseline to after the six-week yoga program. An improvement in scores was also seen for feelings of happiness, positivity, personal satisfaction, and self-confidence from baseline to the end of the intervention. Improvements in the unsubstantiated parameters of patience and fatigue were also observed after the yoga intervention (Figure 2).

Students described favorable effects. Comments received after the study included “I look forward to these evenings because I leave feeling relaxed”… “It clears my mind and helps improve my focus”… “I am more relaxed because I have become more aware of my breath”… “I feel more grounded and have noticed my posture has improved!”.

## 4. Discussion

Our study showed that six weeks of yoga including physical postures, breathing exercises, and meditation may be effective in reducing stress levels and improving aspects of personal wellbeing in medical students prior to taking their exams. Paired *t*-tests showed a statistically significant reduction in perceived stress (18.44 versus 14.52; *p* = 0.004) after the six-week intervention. Similar results have been reported. A pilot study carried out in Montreal evaluated the efficacy of a yoga intervention in first-year female medical students with a mean age of 22 and showed a significant reduction in PSS scores from baseline to the end of the intervention (22.15 versus 13.38) [15].

The results of our self-assessment survey showed a significant improvement in participants' levels of peace, focus, and endurance with an increase in points ranging between one and four from baseline to after six weeks of yoga and meditation. Improvements in happiness, positivity, personal satisfaction, and self-confidence were also seen after the intervention. Although not validated by the Warwick-Edinburgh mental wellbeing scale, the parameters of patience and fatigue also showed improvements after the six-week yoga program. A randomized control study conducted in India observed the effects of a yoga intervention on stress levels in first-year medical students aged eighteen to nineteen prior to taking exams and showed comparable results to our findings. A subjective assessment form incorporating wellness parameters was used as part of their evaluation. Their participants showed a statistically significant improvement in relaxation and calmness, in the ability to concentrate, and in stamina and exhaustion. These findings directly correlated with a greater number of yoga classes attended over time (*p* < 0.01) [16]. Despite similarities, neither of these studies may be generalizable to the American population because of cultural distinctions and variations in the medical training programs.

Limitations of our study include the small number of participants. Invariably, this reduces the power in determining significant differences seen in perceived stress and self-assessment scores from baseline to after the intervention. The absence of a control group did not allow us to discern our participant outcome from one that may have been influenced by other factors such as the natural history of stress and wellbeing over time. On the intake form, one student reported using Wellbutrin and Lexapro for anxiety and depression and another was taking sertraline for OCD management: both preexisting conditions could be confounded with reports of perceived stress.

Of this self-selected group, twenty (74%) students had previously practiced yoga and 11 (41%) had experience with meditation suggesting a possible bias in favor of this mind body intervention. Twenty (85%) students were involved in other regular exercise activities at the beginning of the yoga classes and may have continued this throughout the six-week intervention possibly impacting survey scores. However, considering the sporadic experience of yoga and meditation reported and the notable variation in hours of weekly exercise amongst this group of students, this sample could be representative of a randomly selected population. As this was a pre- and postdesigned study without a control group, the limitations of this study are acknowledged.

## 5. Conclusion

A future study with a larger sample size and separate yoga, exercise, and control groups should be included. In an effort to minimize the potentially detrimental psychological burden on medical students, the addition of a yoga program to the medical school curriculum could be a feasible option. Yoga is inexpensive, potentially rewarding, and safe when taught by experienced certified trainers.
